# Status of diabetes mellitus in different regions of KSA and update on its management

**DOI:** 10.3389/fcdhc.2024.1482090

**Published:** 2024-12-20

**Authors:** Mabrouk AL-Rasheedi, Yasir Alhazmi, Lamees Abdullah AlDaiji, Lamya Abdullah AlDaiji, Fatimah Ismail Mobarki, Khuzama Mohammed Almuhaysini, Jawza Salem Alshammari, Nouf Awadh Almistadi, Saeed Adnan Yoldash, Nouf Almaqwashi, Rawabi Saleh Al Abdulgader, Mohammed Yahya Mashyakhi, Sadin Alamro, Ismail A. Walbi, Khawaja Husnain Haider

**Affiliations:** ^1^ Albukairah General Hospital, Ministry of Health, Albukairah, Saudi Arabia; ^2^ Department of Clinical Pharmacy, College of Pharmacy, Najran University, Najran, Saudi Arabia; ^3^ College of Pharmacy, Qassim University, Buraidah, Saudi Arabia; ^4^ College of Pharmacy, Jazan University, Jizan City, Saudi Arabia; ^5^ Pharmacy Department, Dr. Abdul Rahman Al-Mishari Hospital, Riyadh, Saudi Arabia; ^6^ Diabetic Center, King Abdulaziz Hospital, Jeddah, Saudi Arabia; ^7^ King Abdullah International Medical Research Center (KAIMRC), Ahsa, Eastern Region, Saudi Arabia; ^8^ Pharmacy Department, Najran Armed Forces Hospital, Najran, Saudi Arabia; ^9^ Department of Basic Sciences, College of Medicine, Sulaiman Al Rajhi University, Al-Bukairyah, Saudi Arabia

**Keywords:** complication, diabetes mellitus, KSA, management, prevalence

## Abstract

**Background:**

Complications of diabetes and its associated comorbidities can cause rapid progression of type II diabetes mellitus (T2DM). It comes at high costs and affects a patient’s quality of life. We aim to assess T2DM in KSA, including the demographics, medications, complications, and comorbidities, as it remains an integral part of Vision 2030.

**Methods:**

Observational retrospective study was designed spanning five administrative regions of KSA. A total of 638 patients’ records were randomly selected from general hospitals and diabetes centers from 2017 to 2020, and the collected were statistically analyzed.

**Results:**

Most (77%) selected patients had uncontrolled diabetes, showing a statistically significant correlation between regions and diabetes control. The Northern, Central, and Southern regions had the highest uncontrolled percentage with less than 20% control, while Western and Eastern regions’ control percentages were around 40% of subjects. Eighty percent of the uncontrolled BP patients had uncontrolled diabetes contrasting the 68% of the BP-controlled patients. Biguanides, DPP-4 inhibitors, GLP-1 agonists, Insulin, and SGLT-2 inhibitors are the most common diabetes medications. Metformin was the most prescribed in all regions, followed by DPP4. Results showed that patients used one to four non-diabetes drugs on average. Dispensing of vitamin B complex and statins were higher in diabetes centers than in hospitals. Retinopathy and peripheral neuropathy were the most common complications, while hypertension and ASCVD were the most common comorbidities.

**Conclusion:**

Results showed a poor glycemic control situation in the kingdom that necessitates implementing stricter measures to hinder disease progression and reduce complications and comorbidities. Increasing awareness, training, and monitoring programs with larger sample sizes and broader distribution is highly recommended nationally.

## 1 Introduction

Diabetes mellitus (DM) is one of the leading health issues worldwide, and the number of patients is steadily increasing in developed and developing countries (1). It is a noninfectious chronic disease primarily caused by reduced insulin production or tissue sensitivity to insulin that leads to hyperglycemia which is detrimental to body tissues and organs (2, 3). This non-communicable chronic disorder has a multifactorial etiology involving genetic and environmental factors during its development (4). Due to its chronic nature, the severity of the complications, and the required management strategies, diabetes is an expensive disease affecting patients, their families, and the healthcare system (5). Causes for this widespread global epidemic include population explosion, aging, sedentary lifestyle, and unhealthy eating habits related to obesity (6). The International Diabetes Federation (IDF) has estimated that the total number of persons with DM worldwide will rise from 171 million in 2000 to 366 million by 2030 (7). The number of patients (20-79 years) with DM in the MENA region was 73 million in 2021 and is expected to become 136 million by 2045 (7).

In KSA, DM is quickly reaching disturbing proportions and becoming a significant cause of medical complications and death (5). KSA is among the top ten countries with the highest prevalence of DM and is expected to be among the five countries with the highest majority of type 2 DM (T2DM) in 2030 (8). According to World Health Organization (WHO), KSA ranks second highest in the Middle East after Kuwait and seventh in the world in the rate of diabetes, having nearly 7 million diabetic and 3 million pre-diabetic patients (8, 9), costing around 13.9% of the total health expenditure in KSA (10). However, unlike developed countries, the research conducted in KSA focuses on DM's incidence, prevalence, and socio-demographic properties rather than search for improved health and health-related quality-of-life plans to minimize social and personal expenses spent on diabetes.

Studies on KSA’s T2DM patients are mostly cross-sectional studies. Alomari et al. (2022) reported high prediabetes among the Saudi population in Albaha (11). Alqurashi et al. (2009) found that the prevalence of diabetes is high among the Saudi population, posing a major clinical and public health issue (12). Bahijri et al. (2016) found that the prevalence of diabetes and prediabetes found that age was the strongest predictor of DM and prediabetes, followed by obesity, where 50% of people aged 50 years and over had DM. Another 10–15% had prediabetes in Jeddah (13). Khan et al. (2014) found that the high percentage of chronic complications among diabetic patients showed obesity, hypertension, and dyslipidemia as the most related factors to chronic complications in Alhasa (14).

KSA has transformed the healthcare sector, where diabetes and other uncommunicable diseases are at the heart of the challenges. According to the Health Sector Transformation Program within KSA 2030 vision, it is expected that the number of diabetes patients will reach 8.4 million by 2030, and it is a significant goal to avoid such results (15). The Kingdom has launched the Model of Care project to promote preventive measures and raise awareness of healthcare providers and patients to achieve this goal (16). Developing unified guidelines and training programs for health care providers, in addition to establishing physical and virtual medication counseling to offer detailed information about medications and complications of the disease for diabetes patients, are the primary goals (16, 17). Several initiatives have been launched to improve the quality of patients’ lives and cut the costs related to diabetes treatment in a practical and time-efficient system (15).

The diabetes situation in KSA is poorly documented, resulting in uninformative decisions that may add to the problem (9, 18). Proper documentation of diabetes across KSA should help decision-makers to create strategic plans to minimize the prevalence of the disease, alleviate the patients, avoid medication chaos, and effectively handle the most common complications and comorbidities. The American Diabetes Association (ADA) has set diabetic care standards that are annually revised. However, despite convincing evidence and clear guidelines, these guidelines must be better followed among healthcare providers in KSA.

The present study is divided into two parts, the present article (first part) aims at assessing the situation of T2DM in KSA based on the ADA guidelines, and the second part will determine the commitment of physicians of other specialties and seniorities to the ADA guidelines to alleviate disease complications and comorbidities. After performing the present work in August 2021, Saudi National Diabetes Center developed a guideline to help healthcare providers determine the most appropriate treatment options (19). No contradictions between the Saudi and the ADA Guidelines.

## 2 Methods

An observational retrospective study was conducted to assess the situation of T2DM across KSA, including subjects from general hospitals and diabetes centers. Patients’ data were collected from medical records from 2017 to 2020. Records were selected randomly after a completely randomized design. Five regions were investigated to represent KSA following the administrative regions of the Kingdom (Central, Northern, Southern, Western, and Eastern).

### 2.1 Inclusion and exclusion criteria

Patients (age 20 years or older) diagnosed as T2DM patients for over a year and receiving antihyperglycemics should be treated by primary physicians. Patients younger than 20 years or with gestational diabetes or on dialysis, or treated with more than one physician were excluded from the study.

### 2.2 Study sites

The study was conducted among different tertiary hospitals and diabetes centers across all administrative regions of KSA. Participated general hospitals and diabetes centers are listed in **Table 1**.

**Table 1:** Summarizes the regions, hospitals, and diabetic centers included in the study.

Region	General Hospitals	Diabetes Centers
**Central Region**	-Badayea General Hospital, Qassim. -King Salman Hospital, Riyadh. -Alrass General Hospital, Qassim.	-Diabetes and Endocrine Center, King Saud Hospital, Qassim -Diabetes Center in Unizah, Qassim
**Northern Region**	-Hail General Hospital, Hail -King Khalid Hospital, Hail	-Diabetes and Endocrine Center, King Salman Specialist Hospital, Hail
**Southern Region**	King Fahad Central Hospital, Jazan	-Diabetes and Endocrine Center, Jazan
**Western Region**	-King Abdulaziz Hospital, Jeddah	-Diabetes and Endocrine Center, Jeddah
**Eastern Region**	-Almoosa Specialist Hospital, Al-Ahassa -Dammam Medical Complex, Dammam	Diabetes Center in King Fahad Hospital, Hofuf

### 2.3 Data tools and extraction

Five teams (each comprising 3-5 members) were trained for data collection and entry. An online form was created for such a purpose. All members were healthcare providers recruited from different universities and hospitals. Patients’ data were collected in a designed form that contained several sections:

1. Demographics and medical information of patients and name of hospital or diabetes center and primary physician.
2. All medications are used for diabetes control or other chronic diseases and supplementations.
3. Biochemical test results, complications, and comorbidities of each patient.
4. Treatment plan and seniority and specialty of each primary physician.

Data were collected from all teams and recorded to form the final data set for further analysis.

### 2.4 Sample size

The latest data from Saudi MOH shows that 24% of the population has T2DM (11). According to WHO, the population having T2DM in KSA is around 7 million (11). A total of 680 patient records were collected, and 42 were removed due to a data integrity check, leaving 638 patients for analysis. Data were collected to represent all levels of each demographic variable, including regions, gender, age, and hospital type. The sample size was determined using the RaoSoft tool for sample size calculation (20).

### 2.5 Statistical data analysis and data handling

Data were analyzed using Minitab 20, SPSS 25, and Excel 365. Data was cleansed before running any statistical analyses. Missing data and mistyping errors were checked. Descriptive statistics, including mean, Standard error (SE), standard deviation (SD), minimum (min), First quartile (Q1), median, third Quartile (Q3), and maximum (max), were calculated for quantitative variables. In contrast, the count and percentage of qualitative variables, including levels of each variable, were calculated. Inferential statistics were used to compare results or find correlations among different variables and levels of each variable.

The present study included 15 qualitative and five quantitative variables. All variables were tested for comparisons or correlations about diabetes mellitus. Regions (five levels), hospital type (two levels), gender (two levels), age groups (three levels), diabetes control (two levels), cholesterol control (two levels), triglycerides control (two levels), serum creatinine control (two levels), BP control (two levels), ASCVD status (two levels) diabetes medication (two levels for each drug), other than diabetes medications (two levels for each drug), supplementation (two levels for each drug), complications (two levels for each complication), comorbidities (two levels for each comorbidities). Five quantitative variables investigated included HbA1c, FBS, Cholesterol level, Triglycerides, and Serum creatinine levels.

All FBS, cholesterol, and triglyceride numbers were converted into mg/dl for data analysis. In contrast, serum creatinine in mg/dL was converted to µmol/L to fit the ADA guidelines numbers. HbA1c and FBS were used to classify patients as T2DM-controlled and uncontrolled patients (7% for <60 years and 7.3 for >60 years old patients based on the average of the most recent three patient records), while FBS was used in cases where A1c records were missing at the level of (130 mg/ dl). According to the ADA, BP results were also classified as controlled and uncontrolled patients (<140/90mmHg is the controlled level for all patients except for the ASCVD patients with <130/80 mmHg level). According to the ADA guidelines, the cholesterol control level is < 200 mg/dl and Triglycerides (< 150mg/dl).

The *t2* test was used to investigate correlations among different variables for analyzing qualitative variables. Two independent sample t-tests, One-way and Two-way ANOVA, have been used to correlate qualitative and quantitative variables and compare levels of each qualitative variable about the five quantitative variables. All variables parametric assumptions have been tested, and Box-Cox transformation for non-normal dependent variables was applied whenever needed using the optimal λ method. Different comparisons were made under the fit General linear model menu in Minitab 20. Results showed a good fit for other models, while normal residual probability plots showed a linear attitude for all analyses after data transformation. P values were considered significant at α < 0.05. *Post hoc* analyses of the interactions among all groups were done using the Tukey test for pairwise comparisons. Results of the *post hoc* analyses were represented as letters where groups that shared the same letters were non-significantly different, while different letters expressed significant differences among different groups.

### 2.6 Ethical approval

This study was approved by the Ethical Committee at the Ministry of Health with numbers 1441–1876938.

## 3 Results

### 3.1 Characteristics of the study participants

#### 3.1.1 Demographics and diabetes control

**Figure 1:**
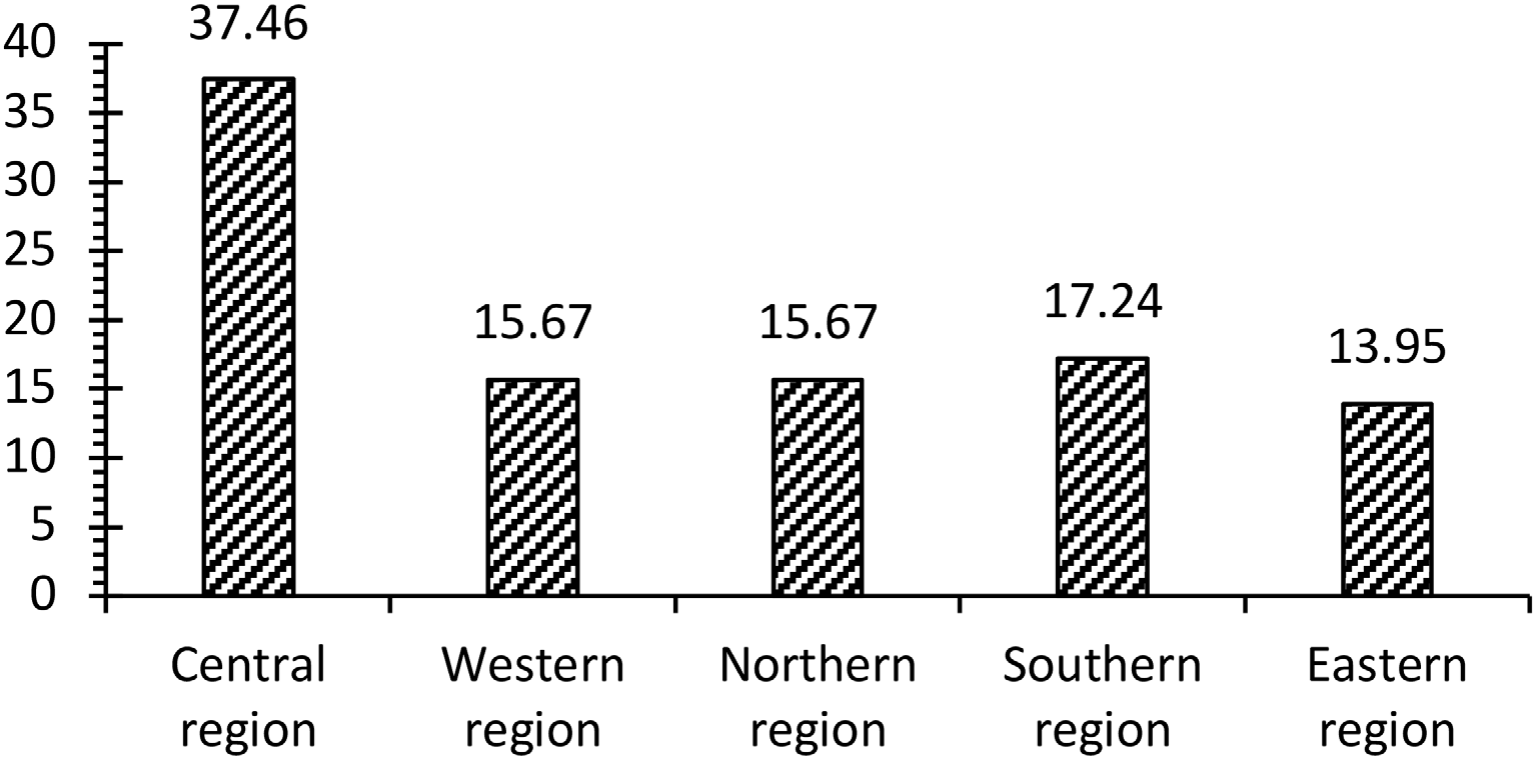
This figure illustrates the proportion of the participants in each region.

The five major administrative regions have been represented proportionately to the population size of each region (**Figure 1**). Genders and Hospital types were represented equally. Age groups showed a higher rate of participants between 40 and 60 years old, with around 50%, followed by older adults over 60, with about 40% of the subjects. A general description of the demographic characteristics of the participants is presented in (**Table 2**).

**Table 2:** Demographic characteristics of the study sample (N= 638).

	Total count (%)	Uncontrolled	Controlled	*X^2^ P*-value
Regions				
Central region	239 (37.46)	194 (81.17)	45 (18.83)	< 0.001**
Western region	100 (15.67)	61 (61)	39 (39)	
Northern region	100 (15.67)	91 (91)	9 (9)	
Southern region	110 (17.24)	88 (80)	22 (20)	
Eastern Region	89 (13.95)	55 (61.8)	34 (38.2)	
Hospital Type				
General Hospital	325 (50.94)	243 (74.77)	82 (25.23)	0.254
Diabetes and Endocrine Centers	313 (49.06)	246 (78.59)	67 (21.41)	
Gender				
Male	326 (51.1)	240 (73.62)	86 (26.38)	0.065
Female	312 (48.9)	249 (79.81)	63 (20.19)	
Age				
20-40 years old	68 (10.66)	48 (70.59)	20 (29.41)	0.186
41-60 years old	317 (49.69)	252 (79.5)	65 (20.5)	
More than 60 years old	253 (39.66)	189 (74.7)	64 (25.3)	

The above table summarizes participant demographics, including the percentage of participants with diabetes control in each region. It also highlights the distribution of participants by age group and gender.

**Highly significant.

**Figure 2:**
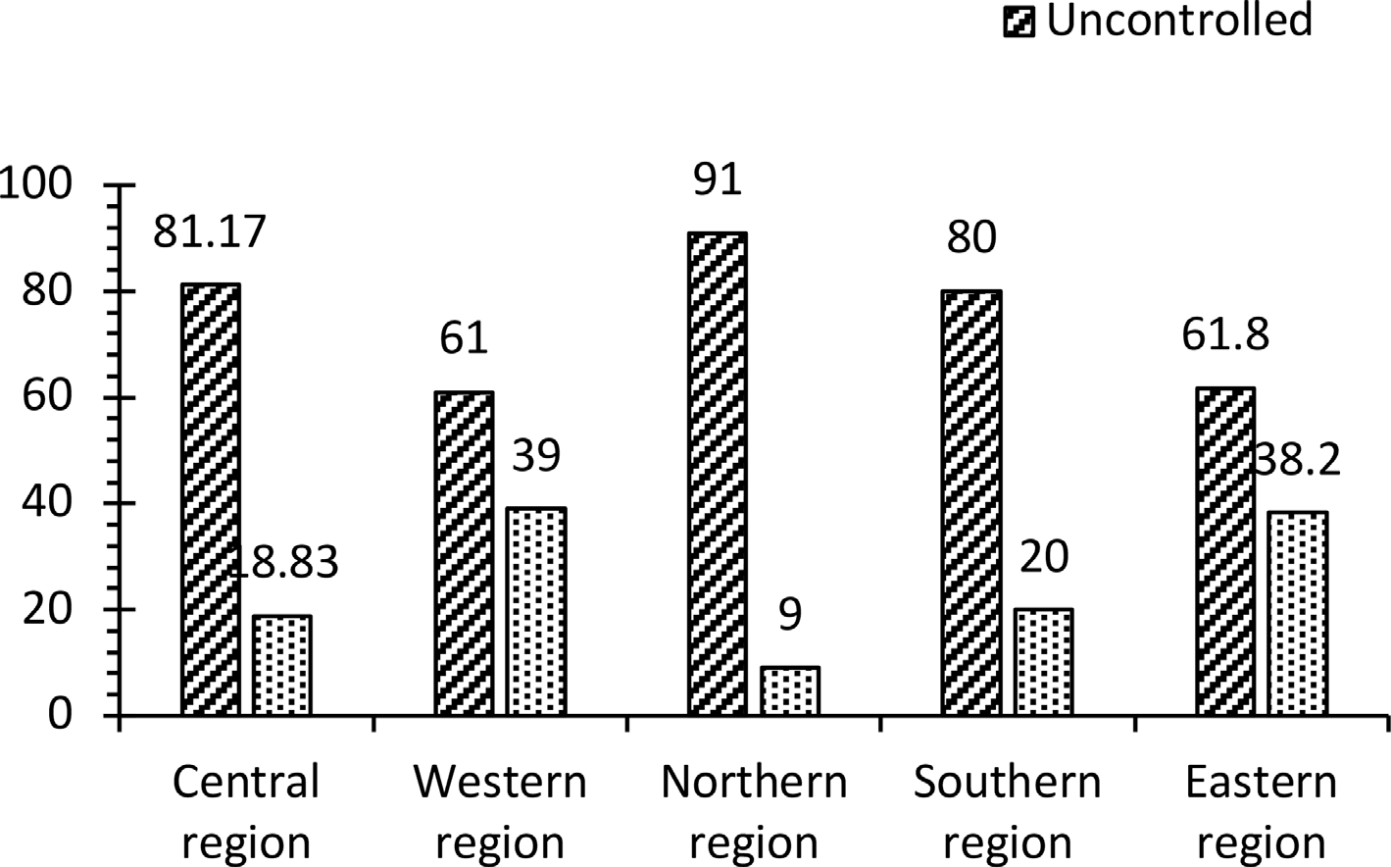
This figure shows the proportion of the participants with controlled and uncontrolled diabetes in each region.

Data showed that 77% of the sample had uncontrolled T2DM and BP, while 23% had an ASCVD. Results showed a statistically significant correlation between the region and T2DM control. The Northern region, followed by the Central and Southern regions, had the lowest controlled T2DM percentage (< 20% control). In comparison, the control percentage of Western and Eastern regions was 40% (**Figure 2**). Results of hospital type, gender, age, and ASCVD status showed no significant correlations between these variables and T2DM control. BP and serum creatinine results showed a significant correlation with diabetes control. Most of the uncontrolled BP patients (80%) had uncontrolled diabetes compared to the BP-controlled patients (68%), while a negative correlation between serum creatinine vs. T2DM management was observed (**Table 2**).

#### 3.1.2 Biochemical indicators

The average body weight was 81 kg (range 41-164 kg), while HbA1c levels varied greatly among the participants (range: 5 to 16.3%) with an average of 8.6%, indicating an uncontrolled T2DM situation. A similar trend was noticed in the FBS results (average 183). Cholesterol, triglycerides, serum creatine levels, and a general description of biochemical indicators of the participants are presented in **Table 3**.

**Table 3:** Patients’ biochemical indicators (N= 683).

Variables	Mean ± SE	SD	Min	Q1	Median	Q3	Max	Range	IQR
**Patient weight**	81.486 ± 0.652	16.461	41	71	82	89	164	123	18
**HbA1c (%)**	8.5975 ± 0.0696	1.7046	5	7.3333	8.48	9.6717	16.3	11.3	2.36
**BP (High)**	137.26 ± 0.873	18.81	92	123.33	134	147	215	123	23.67
**BP (Low)**	75.483 ± 0.481	10.367	48	69	76	81.625	112	64	12.625
**FBS**	183.76 ± 2.73	66.42	59.62	133.58	171.23	225	508.11	448.49	91.42
**TC**	149.87 ± 2.48	62.67	29.52	107.89	150	180.24	654.34	624.82	72.35
**TG**	114.55 ± 3.11	78.64	10.7	58.48	112.27	151.27	940.71	930.01	92.8
**SC**	90.57 ± 3.38	85.3	26.13	60.66	75.63	92.48	1105	1078.87	31.82

BP, Blood pressure; FBS, Fasting blood sugar; HbA1c, Glycated hemoglobin; SC, Serum creatinine; TC, Total cholesterol; TG, Triglyceride.

This table illustrates the participant's biomedical indicators.

### 3.2 Expression of biochemical indicators in subject characteristics

#### 3.2.1 Biochemical indicators about subjects’ characteristics

Our data showed that Eastern and Western regions had the lowest HbA1c and FBS levels. Serum creatine showed higher levels among the male group than the female group and the controlled T2DM group than the uncontrolled group. Results showed a significant correlation between triglycerides and cholesterol levels (**Table 4**).

**Table 4:** Patient’s controlled and uncontrolled some parameters.

	Total count (%)	Uncontrolled	Controlled	*X^2^ P*-value
Cholesterol				
Uncontrolled	90 (14.11)	73 (81.11)	17 (18.89)	0.28
Controlled	548 (85.89)	416 (75.91)	132 (24.09)	
Triglycerides				
Uncontrolled	161 (25.24)	123 (76.4)	38 (23.6)	0.931
Controlled	477 (74.76)	366 (76.73)	111 (23.27)	
Serum creatinine				
Uncontrolled	44 (6.9)	28 (63.64)	16 (36.36)	0.035*
Controlled	594 (93.1)	461 (77.61)	133 (22.39)	
Blood Pressure Control				
Uncontrolled	497 (77.9)	393 (79.07)	104 (20.93)	0.006**
Controlled	141 (22.1)	96 (68.09)	45 (31.91)	
ASCVD				
No	490 (76.8)	369 (75.31)	121 (24.69)	0.146
Yes	148 (23.2)	120 (81.08)	28 (18.92)	
Diabetes Control				
Uncontrolled	489 (76.65)	--	--	--
Controlled	149 (23.35)	--	--	

ASCVD, Atherosclerotic cardiovascular disease.

The above table displays the percentage of participants with controlled and uncontrolled cholesterol, triglyceride, serum creatinine, blood pressure, and diabetes. It also shows the proportion of participants with atherosclerotic cardiovascular disease.

#### 3.2.2 Subject characteristics about each other

The control levels of cholesterol, triglycerides, serum creatinine, and BP, in addition to the presence or absence of ASCVD, showed no correlation except for the cholesterol and triglycerides control, where 78.6% of the controlled group of cholesterol were found to be controlled in triglycerides vs. 51 % in the uncontrolled group (**Table 5**).

**Table 5:** Percentage correlation between parameters.

Cholesterol				Triglycerides			
Blood Pressure	Uncontrolled	Controlled	*P* value	Blood Pressure	Uncontrolled	Controlled	*P* value
Uncontrolled	75.6%	78.3%	0.563	Uncontrolled	80.7%	76.9%	0.314
Controlled	24.4%	21.7%		Controlled	19.3%	23.1%	
Triglycerides				Serum creatinine			
Uncontrolled	48.9%	21.4%	.000^*^	Uncontrolled	9.3%	6.1%	0.161
Controlled	51.1%	78.6%		Controlled	90.7%	93.9%	
Serum creatinine				ASCVD			
Uncontrolled	10.0%	6.4%	0.210	No	77.6%	76.5%	0.771
Controlled	90.0%	93.6%		Yes	22.4%	23.5%	
ASCVD				Serum Creatinine			
No	84.4%	75.5%	0.064	Blood Pressure	Uncontrolled	Controlled	*P* value
Yes	15.6%	24.5%		Uncontrolled	75.0%	78.1%	0.631
ASCVD				Controlled	25.0%	21.9%	
Blood Pressure	No	Yes	*P* value	ASCVD			
Uncontrolled	78.2%	77.0%	0.770	No	65.9%	77.6%	0.076
Controlled	21.8%	23.0%		Yes	34.1%	22.4%	

ASCVD, Atherosclerotic cardiovascular disease.

This table shows the correlation between the control of cholesterol, triglycerides, serum creatinine, and blood pressure, as well as the presence or absence of atherosclerotic disease.

### 3.3 Diabetes control and subject characteristics

#### 3.3.1 Diabetes control and variability

**Figure 3:**
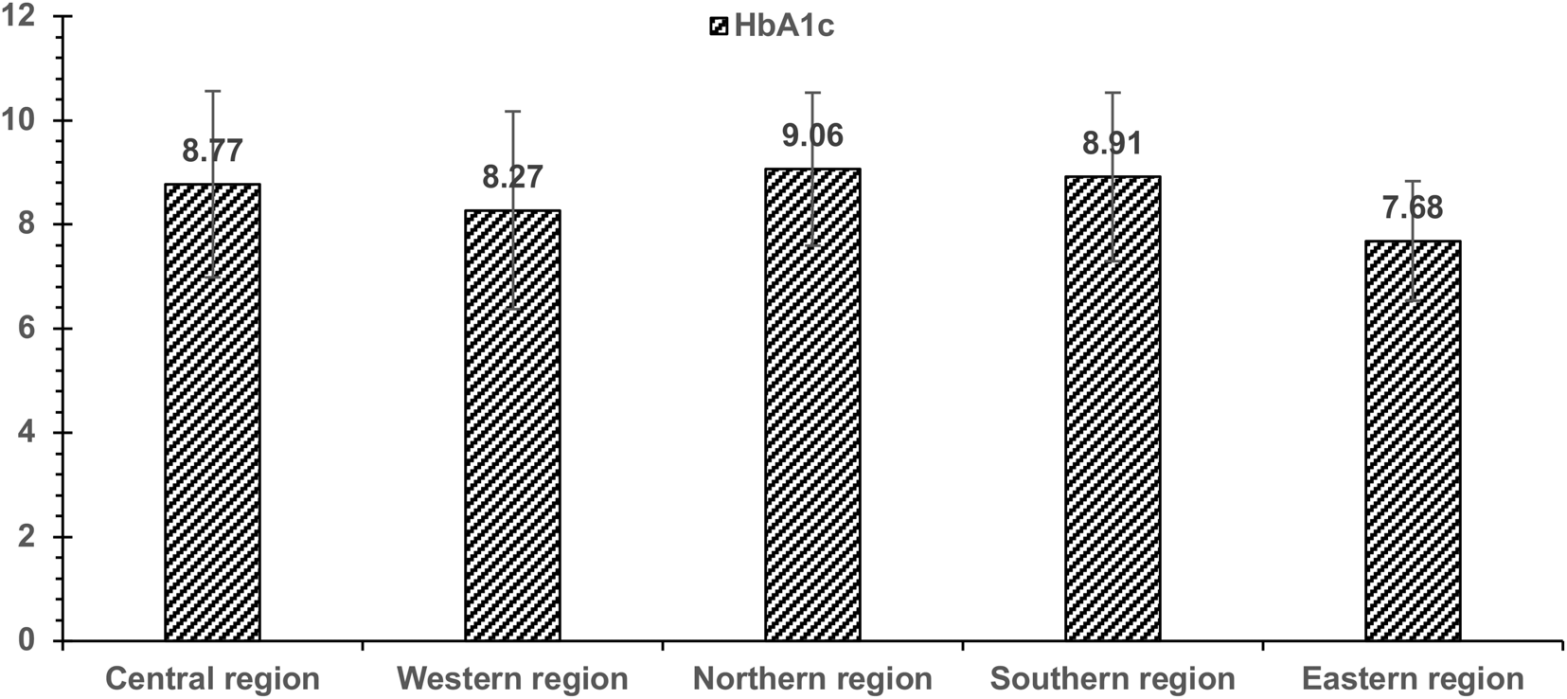
This figure illustrates the levels of HbA1c across regions.

HbA1c and FBS results showed the lowest average values in Western and Eastern regions vs. other regions (**Figure 3**). HbA1c results of the serum creatinine-uncontrolled group showed lower values than controlled and uncontrolled groups. Cholesterol and triglycerides showed the lowest values in the Northern region. Cholesterol and triglyceride levels were higher in general hospital patients than in diabetes centers. Triglycerides levels were lower in the cholesterol-controlled group, irrespective of the controlled or uncontrolled in diabetes levels (**Table 6**).

**Table 6:** Level of the parameters in regions, hospitals and diabetic centres.

Regions	HbA1c	FBS	Chl	TG	SC
Central region	8.77 ± 1.79^AB^	187.13 ± 66.93^AB^	151.59 ± 56.39^B^	117.51 ± 90.82^B^	89.89 ± 68.81^A^
Western region	8.27 ± 1.9^BC^	163.42 ± 65.21^C^	167.21 ± 42.39^AB^	126.83 ± 63.05^AB^	105.26 ± 159.27^A^
Northern region	9.06 ± 1.47^A^	206.99 ± 60.78^A^	102.23 ± 86.34^C^	39.84 ± 30.51^C^	83.23 ± 49.17^A^
Southern region	8.91 ± 1.62^A^	185.28 ± 62.92^ABC^	174.15 ± 58.45^A^	143.06 ± 71.98^A^	82.41 ± 79.05^A^
Eastern Region	7.68 ± 1.15^C^	169.43 ± 68.45^BC^	149.26 ± 39.04^B^	141.52 ± 47.27^AB^	94.2 ± 21.54^A^
Hospital Type					
General Hospital	8.41 ± 1.58^B^	184.55 ± 68.33^A^	134.74 ± 57.51^B^	100.48 ± 66.39^B^	92.19 ± 64.64^A^
Diabetes Centers	8.78 ± 1.8^A^	183.04 ± 64.7^A^	165.57 ± 64.01^A^	129.16 ± 87.34^A^	88.89 ± 102.53^A^
Gender					
Male	8.49 ± 1.77^A^	178.87 ± 64.54^A^	147.58 ± 56.75^A^	116.64 ± 82.69^A^	105.64 ± 106.79^A^
Female	8.7 ± 1.63^A^	188.69 ± 68^A^	152.27 ± 68.32^A^	112.37 ± 74.25^A^	74.82 ± 49.99^B^
Age					
20-40 years old	8.62 ± 1.99^A^	186.36 ± 74.06^A^	157.29 ± 64.01^AB^	121 ± 81.46^A^	74.04 ± 26.53^A^
41-60 years old	8.64 ± 1.65^A^	184.46 ± 63.19^A^	155.8 ± 67.71^A^	118.22 ± 84.67^A^	93.22 ± 99.98^A^
More than 60 years old	8.53 ± 1.69^A^	182.27 ± 68.58^A^	140.44 ± 54.23^B^	108.23 ± 69.36^A^	91.69 ± 74.74^A^
Diabetes control					
Uncontrolled	9.27 ± 1.39^A^	202.01 ± 63.82^A^	149.63 ± 67.51^A^	112.6 ± 82.28^A^	86.74 ± 76.98^B^
Controlled	6.55 ± 0.54^B^	126.48 ± 33.92^B^	150.65 ± 43.29^A^	120.94 ± 65.15^A^	103.14 ± 107.57^A^
Cholesterol					
Uncontrolled	9.17 ± 1.99^A^	187.25 ± 67.33^A^	249.87 ± 79.98^A^	176.1 ± 124.3^A^	92.7 ± 83.71^A^
Controlled	8.50 ± 1.63^B^	183.15 ± 66.3^A^	133.45 ± 40.21^B^	104.44 ± 62.95^B^	90.22 ± 85.63^A^
Triglycerides					
Uncontrolled	8.56 ± 1.66^A^	189.72 ± 69.25^A^	177.43 ± 52.85^A^	212.41 ± 82.88^A^	101.89 ± 119.01^A^
Controlled	8.61 ± 1.72^A^	181.58 ± 65.29^A^	140.57 ± 63.03^B^	81.52 ± 40.44^B^	86.75 ± 70.13^A^
Serum creatinine					
Uncontrolled	7.77 ± 1.45^B^	158.18 ± 60.29^B^	141.1 ± 76.8^A^	115.9 ± 74.4^A^	297 ± 234.3^A^
Controlled	8.66 ± 1.71^A^	185.67 ± 66.51^A^	150.52 ± 61.52^A^	114.45 ± 79^A^	75.28 ± 20.99^B^
Blood Pressure Control					
Uncontrolled	8.64 ± 1.67^A^	186.3 ± 66.48^A^	147.9 ± 62.81^A^	115.19 ± 82.93^A^	92.28 ± 89.56^A^
Controlled	8.44 ± 1.83^A^	174.46 ± 65.6^A^	156.82 ± 61.87^A^	112.29 ± 61.37^A^	84.55 ± 68.16^A^
ASCVD					
Yes	8.58 ± 1.72^A^	184.42 ± 66.63^A^	153.7 ± 65.22^A^	115.91 ± 82.35^A^	87.08 ± 80.13^A^
No	8.66 ± 1.67^A^	181.37 ± 65.85^A^	137.18 ± 51.53^B^	110.04 ± 64.89^A^	102.13 ± 100^A^

ASCVD, Atherosclerotic cardiovascular disease; FBS, Fasting blood sugar; HbA1c, Glycated hemoglobin; SC, Serum creatinine; TC, Total cholesterol; TG, Triglyceride.

^A, B, C^Groups with the different letters are significantly different.

The table above shows the levels of fasting blood sugar, HbA1c, cholesterol, triglycerides, and serum creatinine across various regions, hospitals, and diabetes centers. It also illustrates these levels in relation to age, gender, both controlled and uncontrolled diabetes, blood pressure, and the presence or absence of atherosclerotic disease.

#### 3.3.2 Gender variability

Results showed that both male and female groups of the Eastern region had the lowest HbA1c and FBS levels, while the females of the Northern group had the highest average HbA1c and FBS. Both males and females in the uncontrolled cholesterol group showed higher HbA1c and FBS. Cholesterols and triglycerides showed the lowest levels in the Northern region. Serum creatinine results were more elevated in all males within all variables than in females (**Table 7**).

**Table 7:** Controlled and uncontrolled of some parameters across regions and hospitals.

Regions	Control	HbA1c	FBS	Chl	TG	SC
Central Region	Uncon	9.41 ± 1.46^A^	202.47 ± 64.29^AB^	151.17 ± 57.51^B^	117.73 ± 94.52^A^	88.51 ± 72.17^A^
	Con	6.5 ± 0.6^C^	124.25 ± 31.61^C^	153.42 ± 51.84^AB^	116.6 ± 73.7^A^	95.88 ± 52.15^A^
Western Region	Uncon	9.43 ± 1.5^A^	190.94 ± 64.58^AB^	172.65 ± 46.91^AB^	129.72 ± 72.33^A^	96 ± 138.3^A^
	Con	6.45 ± 0.56^C^	120.37 ± 37.11^C^	158.7 ± 32.93^AB^	122.3 ± 45.41^A^	119.8 ± 188.5^A^
Northern region	Uncon	9.34 ± 1.22^A^	213.44 ± 58.56^A^	103.97 ± 90.3^D^	40.94 ± 31.67^B^	81.37 ± 42.76^A^
	Con	6.3 ± 0.56^C^	141.8 ± 43.4^BC^	84.64 ± 12.17^CD^	28.67 ± 9.04^B^	102 ± 94.4^A^
Southern region	Uncon	9.43 ± 1.4^A^	198.49 ± 60.6^AB^	177.27 ± 61.51^A^	145.26 ± 67.83^A^	79.14 ± 78.46^A^
	Con	6.91 ± 0.32^C^	133.69 ± 42.52^C^	161.68 ± 43.05^AB^	134.3 ± 87.9^A^	95.5 ± 81.9^A^
Eastern region	Uncon	8.36 ± 0.9^B^	198.7 ± 74.8^AB^	150 ± 43.28^ABC^	141.85 ± 51.56^A^	91.28 ± 21.46^A^
	Con	6.59 ± 0.45^C^	127.68 ± 21.58^C^	148.08 ± 31.56^ABC^	140.98 ± 40.11^A^	98.92 ± 21.13^A^
Hospital Type						
General Hospital	Uncon	9.11 ± 1.27^B^	203.93 ± 67.36^A^	134 ± 61.71^B^	97.68 ± 69.17^B^	88.59 ± 65.47^A^
	Con	6.62 ± 0.54^C^	131.49 ± 34.68^B^	136.96 ± 42.94^B^	108.76 ± 56.96^AB^	102.83 ± 61.26^A^
Diabetes Centers	Uncon	9.42 ± 1.48^A^	200.35 ± 60.7^A^	165.08 ± 69.53^A^	127.34 ± 91.2^A^	84.91 ± 86.97^A^
	Con	6.47 ± 0.53^C^	120.78 ± 32.36^B^	167.4 ± 37.72^A^	135.85 ± 71.6^A^	103.5 ± 146.09^A^
Gender						
Male	Uncon	9.26 ± 1.51^A^	196.97 ± 63.83^A^	148.58 ± 61.75^A^	114.98 ± 90.25^A^	99.52 ± 93.95^AB^
	Con	6.62 ± 0.52^B^	131.39 ± 36.12^B^	144.77 ± 39.75^A^	121.28 ± 56.66^A^	122.7 ± 135.61^A^
Female	Uncon	9.29 ± 1.28^A^	206.63 ± 63.6^A^	150.64 ± 72.74^A^	110.32 ± 73.89^A^	74.42 ± 53.34^C^
	Con	6.46 ± 0.55^B^	119.87 ± 29.73^B^	158.68 ± 46.85^A^	120.48 ± 75.7^A^	76.43 ± 33.95^BC^
Age						
20-40 years old	Uncon	9.52 ± 1.69^A^	217.42 ± 65.88^A^	160.99 ± 72.76^AB^	119.1 ± 89.27^A^	70.79 ± 24.83^A^
	Con	6.53 ± 0.58^B^	111.46 ± 15.98^B^	148.42 ± 35.12^AB^	125.55 ± 60.46^A^	81.84 ± 29.44^A^
41-60 years old	Uncon	9.23 ± 1.37^A^	199.81 ± 61.19^A^	156.17 ± 72.51^A^	116.87 ± 88.49^A^	88.48 ± 80.69^A^
	Con	6.57 ± 0.51^B^	129.07 ± 31.37^B^	154.38 ± 44.86^AB^	123.43 ± 68.18^A^	111.59 ± 152.95^A^
More than 60 years old	Uncon	9.27 ± 1.33^A^	201.31 ± 66.53^A^	138.03 ± 57.11^B^	105.27 ± 70.95^A^	88.47 ± 80.4^A^
	Con	6.54 ± 0.56^B^	127.92 ± 39.02^B^	147.56 ± 44.29^AB^	116.97 ± 64.15^A^	101.2 ± 54.16^A^
Cholesterol						
Uncontrolled	Uncon	9.8 ± 1.69^A^	201.12 ± 66.54^A^	255.8 ± 87.4^A^	168.2 ± 130.2^A^	89.5 ± 88.3^A^
	Con	6.62 ± 0.58^C^	129.34 ± 29.32^B^	224.56 ± 18.55^A^	210.2 ± 90.2^A^	106.6 ± 60^A^
Controlled	Uncon	9.18 ± 1.31^B^	202.18 ± 63.39^A^	131.01 ± 41.28^B^	102.85 ± 66.19^B^	86.26 ± 74.92^A^
	Con	6.55 ± 0.53^C^	126.09 ± 34.58^B^	141.13 ± 35.71^B^	109.45 ± 51.32^B^	102.69 ± 112.39^A^
Triglycerides						
Uncontrolled	Uncon	9.17 ± 1.44^A^	207.38 ± 68.35^A^	178.53 ± 54.95^A^	214.7 ± 89.31^A^	97.6 ± 113.6^A^
	Con	6.67 ± 0.5^B^	133.48 ± 32.5^B^	173.87 ± 45.89^A^	204.97 ± 57.65^A^	115.7 ± 135.9^A^
Controlled	Uncon	9.31 ± 1.37^A^	200.03 ± 62.06^A^	139.92 ± 68.62^B^	78.29 ± 41.12^B^	83.08 ± 59.65^A^
	Con	6.52 ± 0.55^B^	123.94 ± 34.22^B^	142.7 ± 39.54^B^	92.18 ± 36.31^B^	98.85 ± 96.36^A^
Serum Creatinine						
Uncontrolled	Uncon	8.68 ± 1.09^A^	171.5 ± 63.4^B^	132.9 ± 79.4^A^	105.3 ± 69.8^A^	293.8 ± 230.2^A^
	Con	6.41 ± 0.62^B^	137.4 ± 50^BC^	155.4 ± 72.3^A^	134.5 ± 80.8^A^	302.6 ± 248.8^A^
Controlled	Uncon	9.31 ± 1.4^A^	203.81 ± 63.46^A^	150.64 ± 66.68^A^	113.05 ± 83.01^A^	74.16 ± 20.17^B^
	Con	6.57 ± 0.53^B^	125.1 ± 31.33^C^	150.07 ± 38.79^A^	119.31 ± 63.19^A^	79.15 ± 23.28^B^
Blood Pressure Control						
Uncontrolled	Uncon	9.24 ± 1.37^A^	202.7 ± 63.8^A^	147.94 ± 67.41^A^	113.32 ± 86.15^A^	87.9 ± 77.09^A^
	Con	6.55 ± 0.54^B^	127.21 ± 35.02^B^	147.74 ± 41.33^A^	122.26 ± 69.32^A^	108.83 ± 125.13^A^
Controlled	Uncon	9.42 ± 1.47^A^	199.04 ± 64.21^A^	156.55 ± 67.85^A^	109.67 ± 64.3^A^	82 ± 76.77^A^
	Con	6.57 ± 0.54^B^	124.7 ± 31.46^B^	157.39 ± 47.33^A^	117.89 ± 54.88^A^	89.98 ± 44.9^A^
ASCVD						
Yes	Uncon	9.28 ± 1.4^A^	204.3 ± 63.23^A^	153.19 ± 71.25^A^	113.2 ± 86.75^A^	84.47 ± 76.46^B^
	Con	6.57 ± 0.54^B^	126.15 ± 34.45^B^	155.26 ± 41.99^A^	124.19 ± 66.82^A^	95.02 ± 90.28^AB^
No	Uncon	9.25 ± 1.36^A^	194.33 ± 65.51^A^	138.69 ± 53.2^A^	110.77 ± 66.94^A^	93.71 ± 78.48^AB^
	Con	6.49 ± 0.53^B^	128.01 ± 31.92^B^	130.72 ± 43.93^A^	106.88 ± 56.28^A^	138.2 ± 160.1^A^

ASCVD, Atherosclerotic cardiovascular disease; Con, controlled; FBS, Fasting blood sugar; HbA1c, Glycated hemoglobin; SC, Serum creatinine; TC, Total cholesterol; TG, Triglyceride; Uncon, Uncontrolled.

^A, B, C, D*^Groups with the different letters are significantly different.

The above table displays the controlled and uncontrolled levels of various parameters (fasting blood sugar, HbA1c, cholesterol, triglycerides, and serum creatinine) across regions, hospital types, age groups, blood pressure, and the presence or absence of atherosclerotic disease.

#### 3.3.3 Age groups

All age groups in the Eastern region had the lowest HbA1c and FBS, while all the Northern groups had the highest HbA1c and FBS levels. Triglycerides and cholesterol were lower in the Southern regions for all age groups (**Table 7**).

### 3.4 Medications

All medications prescribed for less than ten sample persons were excluded from our results.

#### 3.4.1 Diabetes medication

Around 33% of participants were getting three drugs, followed by 23% for one and two drugs each, while less than 1% were getting five diabetes drugs. The most common diabetes drugs were Biguanides (Metformin), DPP-4 inhibitors, GLP-1 agonists, Insulin, SGLT-2 inhibitors, and Sulfonylurea. A significant correlation was observed between regions and hospital types for almost all prescribed drugs. The Western region had the highest use of metformin (84%), while the Northern region had the lowest. Data showed that DPP-4 inhibitors were much more prescribed in diabetes centers than in general hospitals, while around 62% of patients with ASCVD are not taking Metformin. GLP 1 agonist is mainly used in Western and Eastern regions.

Results showed that 73% of the diabetes-controlled group didn’t take insulin, while 66.5% of the diabetes-uncontrolled group and 67% of ASCVD were on insulin, vs. 71% in the Southern region. SGLT-2 inhibitors were much prescribed in the Western region, and among all regions, it was much more prescribed in general hospitals than in diabetes centers. Results showed that sulfonylurea was less used in the Eastern region, and among all regions, it was much more prescribed in general hospitals than in diabetes centers (**Table 8**).

**Table 8:** Diabetic medications used by participants.

Diabetes medication		Total	T2DM Control		Region					Gender		Hospital Type		Age			BP Control		ASCVD		DM	R	G	T	A	BP	D
			UnCon	Con	CR	WR	NR	SR	ER	M	F	H	DC	20-41	41-60	>60	UnCon	Con	No	Yes	* Denotes P value < 0.05						
		Total	489	149	239	100	100	110	89	326	312	325	313	68	317	253	497	141	490	148							
Biguanides (Metformin)	No	152	23.9	23.5	28.5	16.0	30.0	19.1	19.1	23.0	24.7	26.8	20.8	32.4	23.0	22.5	23.1	26.2	23.5	25.0		*					
	Yes	486	76.1	76.5	71.5	84.0	70.0	80.9	80.9	77.0	75.3	73.2	79.2	67.6	77.0	77.5	76.9	73.8	76.5	75.0							
DPP-4 inhibitors	No	335	52.4	53.0	56.1	43.0	48.0	58.2	51.7	52.8	52.2	60.6	44.1	61.8	50.5	52.6	50.7	58.9	49.8	61.5				*			*
	Yes	303	47.6	47.0	43.9	57.0	52.0	41.8	48.3	47.2	47.8	39.4	55.9	38.2	49.5	47.4	49.3	41.1	50.2	38.5							
GLP-1 agonists	No	567	88.3	90.6	94.6	70.0	88.0	96.4	86.5	89.6	88.1	92.3	85.3	86.8	86.8	92.1	88.7	89.4	89.6	86.5		*		*			
	Yes	71	11.7	9.4	5.4	30.0	12.0	3.6	13.5	10.4	11.9	7.7	14.7	13.2	13.2	7.9	11.3	10.6	10.4	13.5							
Insulin	No	273	33.5	73.2	41.4	45.0	42.0	29.1	61.8	48.2	37.2	45.8	39.6	42.6	41.0	45.1	42.7	43.3	45.7	33.1	*	*	*				*
	Yes	365	66.5	26.8	58.6	55.0	58.0	70.9	38.2	51.8	62.8	54.2	60.4	57.4	59.0	54.9	57.3	56.7	54.3	66.9							
SGLT-2 inhibitors	No	510	78.5	84.6	90.4	54.0	98.0	75.5	66.3	81.3	78.5	83.7	76.0	80.9	78.9	81.0	81.3	75.2	80.6	77.7		*		*			
	Yes	128	21.5	15.4	9.6	46.0	2.0	24.5	33.7	18.7	21.5	16.3	24.0	19.1	21.1	19.0	18.7	24.8	19.4	22.3							
Sulfonylurea	No	412	63.0	69.8	61.9	58.0	58.0	70.9	78.7	61.3	67.9	69.2	59.7	64.7	67.8	60.5	62.2	73.0	63.1	69.6		*		*		*	
	Yes	226	37.0	30.2	38.1	42.0	42.0	29.1	21.3	38.7	32.1	30.8	40.3	35.3	32.2	39.5	37.8	27.0	36.9	30.4							
Number of Medications	M	4	0.6	0.7	1.7	0.0	0.0	0.0	0.0	0.6	0.6	0.9	0.3	0.0	0.9	0.4	0.8	0.0	0.8	0.0	*	*		*			
	1	147	19.2	35.6	31.0	8.0	24.0	12.7	30.3	23.0	23.1	30.2	15.7	32.4	21.8	22.1	21.1	29.8	22.4	25.0							
	2	154	22.7	28.9	22.2	17	29	31.8	22.5	26.1	22.1	26.5	21.7	22.1	21.8	27.7	24.1	24.1	23.5	26.4							
	3	212	35.6	25.5	28.9	35	35	43.6	28.1	33.4	33.0	31.1	35.5	29.4	36.6	30.0	34.2	29.8	35.3	26.4							
	4	106	19.22	8.05	15.90	32	11	10	15.73	13.50	19.87	10.15	23.32	13.24	16.09	18.18	17.51	13.48	15.71	19.59							
	5	15	2.66	1.34	0.42	8	1	1.82	3.37	3.37	1.28	1.23	3.51	2.94	2.84	1.58	2.21	2.84	2.24	2.70							

ASCVD, Atherosclerotic cardiovascular disease; BP, Blood pressure; Con, controlled; CR, Central region; DC, Diabetes center; DM, Diabetes mellitus; DPP-4 inhibitors, Dipeptidyl-peptidase-4 inhibitors; ER, Eastern region; GLP-1 agonists, glucagon-like peptide-1 agonist; H, Hospital; NR, Northern region; SGLT-2 inhibitors, Sodium-glucose co-transporter-2 inhibitors; SR, Southern region; Uncon, Uncontrolled; WR, Western region.

This table illustrates the types of diabetes medications used by participants, along with the proportion of each medication type in diabetes control, region, gender, age, hospital type, control blood pressure, and the presence or absence of atherosclerotic disease.

#### 3.4.2 Drugs other than diabetes drugs

The number of non-diabetic medications ranged between 0 to 10 for each participant, with the largest share of drugs between 0 to 4. Common drugs include alpha-blockers, Antidepressant., Corticosteroids, fenofibrate., NSIAD, Anticoagulants (i.e., Warfarin), Anti-convulsant (i.e., Phenytoin), Antiplatelets (i.e., Aspirin, Clopidogrel), B-blockers, ACEI/ARB, CCB, Diuretics, Levothyroxine, PPI/H2RA (i.e., Omeprazole, Ranitidine), and Statins (i.e., Atorvastatin). Medicines taken by less than ten patients were excluded from the analysis, such as Antibiotics, Anticholinergics, Antispasmodic, Carbonic anhydrase inhibitors, Laxatives, muscle relaxants, Vasodilators, 5-alpha reductase inhibitors, allopurinol, Antacids, evolocumab, Eye drops, Cardiac glycoside, methyl dopa, Nitrates, Xanthine oxidase inhibitor.

A significant correlation was observed between regions, hospital types, age groups, and medications. Data showed that around 45% of T2DM patients took ACEI or ARB, with 57% in the Western region followed by 50% in the Eastern region. About the age, 47% of patients aged between 41-60 years had ACEI or ARB, while only 10% of patients aged between 20-40 years were on ACEI or ARB. On the contrary, ACE or ARB was prescribed for 60% of ASCVD patients vs. 38% in no ASCVD patients.

Beta-blockers were the most prescribed (22%) for patients over 60 years, while 13% were between 40 and 46 years old. Beta-blocker prescription was 18% for uncontrolled BP patients and 8% for controlled patients. On the contrary, 33% of patients with ASCVD used beta blockers vs. 10% of non-ASCVD patients.

Results showed that 32% of Central region patients were on CCB, while the Southern region showed the lowest rate (16%). About age, 32% of patients over 60 were taking CCB, while 10% were in the younger age group (20-40 years). CCBs were prescribed for 31% of ASCVD patients, while 22% for no ASCVD patients.

Statins were prescribed for over 50% of patients; the western region has the highest percentage (89%). In comparison, in only 40% of the Southern region, Statins were more prescribed for females than males, with a 12% difference. Statins’ prescription was higher in diabetes centers (72%) vs. general Hospitals (51%). Statins prescription was higher in the 41-60 age group (64%) vs. 20-41 age group (33%). Uncontrolled BP patients have taken Statins with a 10% difference from the controlled BP patients. ASCVD patients were higher in statin use (74%) vs. the non- ASCVD group (57%).

Diuretics were prescribed to 31% of Western patients, followed by 18% in the Central region. Diuretics were prescribed for patients over 60 and those between 40 and 60. Diuretics were prescribed double folds among ASCVD patients.

Levothyroxine prescription was higher in the Northern and Western regions, with 13% relative to the other regions. Diabetic centers were more elevated than general hospitals in dispensing levothyroxine for their patients. The 20-40 age group used levothyroxine more than other age groups.

Antiplatelet medications positively correlated with age, especially when patients had ASCVD (71% among all regions). Data showed that the use of Warfarin was much more in the Northern region, followed by the Central region (10% and 7.5%, respectively) vs. the Western region with no prescription record. 10% of ACSVD patients get warfarin, while only 2% of non-ASCVD patients get the medication. Phenytoin was not prescribed in the Southern region for diabetes patients. No records of antidepressants or corticosteroids were recorded in the Western region (**Table 9**).

**Table 9:** Non-diabetic medications used by participants.

Non-Diabetes medication		Total	DM Control		Region					Gender		Hospital Type		Age			BP Control		ASCVD		DM	R	G	T	A	BP	D
			UnCon	Con	CR	WR	NR	SR	ER	M	F	H	DC	20-41	41-60	>60	UnCon	Con	No	Yes	* denotes P value < 0.05						
		Total	489	149	239	100	100	110	89	326	312	325	313	68	317	253	497	141	490	148							
Alpha-blockers	No	614	96.93%	93.96%	96.65%	100.00%	90.00%	99.09%	94.38%	92.94%	99.68%	94.15%	98.40%	98.53%	97.16%	94.47%	95.57%	98.58%	95.92%	97.30%		*	*	*			
	Yes	24	3.07%	6.04%	3.35%	0.00%	10.00%	0.91%	5.62%	7.06%	0.32%	5.85%	1.60%	1.47%	2.84%	5.53%	4.43%	1.42%	4.08%	2.70%							
Anticoagulants (Warfarin…)	No	609	95.30%	95.97%	92.47%	100.00%	90.00%	100.00%	98.88%	96.32%	94.55%	93.54%	97.44%	98.53%	95.58%	94.47%	95.77%	94.33%	97.14%	89.86%		*		*			*
	Yes	29	4.70%	4.03%	7.53%	0.00%	10.00%	0.00%	1.12%	3.68%	5.45%	6.46%	2.56%	1.47%	4.42%	5.53%	4.23%	5.67%	2.86%	10.14%							
Anti-convulsant (Phenytoin….)	No	611	95.91%	95.30%	95.40%	92.00%	94.00%	100.00%	97.75%	96.32%	95.19%	95.38%	96.17%	97.06%	96.53%	94.47%	95.77%	95.74%	95.92%	95.27%		*					
	Yes	27	4.09%	4.70%	4.60%	8.00%	6.00%	0.00%	2.25%	3.68%	4.81%	4.62%	3.83%	2.94%	3.47%	5.53%	4.23%	4.26%	4.08%	4.73%							
Antiplatelets (Aspirin, Clopidogrel…)	No	334	51.53%	55.03%	53.56%	47.00%	49.00%	47.27%	65.17%	52.76%	51.92%	51.08%	53.67%	86.76%	52.68%	42.69%	53.12%	49.65%	59.39%	29.05%					*		*
	Yes	304	48.47%	44.97%	46.44%	53.00%	51.00%	52.73%	34.83%	47.24%	48.08%	48.92%	46.33%	13.24%	47.32%	57.31%	46.88%	50.35%	40.61%	70.95%							
B-blockers	No	536	83.23%	86.58%	84.52%	85.00%	84.00%	88.18%	76.40%	83.74%	84.29%	84.00%	84.03%	97.06%	86.75%	77.08%	81.89%	91.49%	89.18%	66.89%					*	*	*
	Yes	102	16.77%	13.42%	15.48%	15.00%	16.00%	11.82%	23.60%	16.26%	15.71%	16.00%	15.97%	2.94%	13.25%	22.92%	18.11%	8.51%	10.82%	33.11%							
ACEI/ARB	No	361	56.03%	58.39%	56.49%	43.00%	67.00%	65.45%	49.44%	55.21%	58.01%	59.69%	53.35%	79.41%	52.37%	55.73%	51.91%	73.05%	61.43%	40.54%		*			*	*	*
	Yes	277	43.97%	41.61%	43.51%	57.00%	33.00%	34.55%	50.56%	44.79%	41.99%	40.31%	46.65%	20.59%	47.63%	44.27%	48.09%	26.95%	38.57%	59.46%							
CCB	No	480	76.48%	71.14%	67.78%	79.00%	73.00%	83.64%	83.15%	76.38%	74.04%	76.62%	73.80%	89.71%	78.23%	67.59%	73.84%	80.14%	77.35%	68.24%		*			*		*
	Yes	158	23.52%	28.86%	32.22%	21.00%	27.00%	16.36%	16.85%	23.62%	25.96%	23.38%	26.20%	10.29%	21.77%	32.41%	26.16%	19.86%	22.65%	31.76%							
Diuretics	No	520	81.80%	80.54%	81.17%	69.00%	85.00%	89.09%	83.15%	82.82%	80.13%	83.08%	79.87%	95.59%	85.17%	73.12%	80.89%	83.69%	84.69%	70.95%		*			*		*
	Yes	118	18.20%	19.46%	18.83%	31.00%	15.00%	10.91%	16.85%	17.18%	19.87%	16.92%	20.13%	4.41%	14.83%	26.88%	19.11%	16.31%	15.31%	29.05%							
Levothyroxine	No	576	92.84%	81.88%	89.96%	87.00%	85.00%	97.27%	92.13%	92.64%	87.82%	92.00%	88.50%	89.71%	90.22%	90.51%	89.74%	92.20%	89.39%	93.24%		*		*	*		
	Yes	62	7.16%	18.12%	10.04%	13.00%	15.00%	2.73%	7.87%	7.36%	12.18%	8.00%	11.50%	10.29%	9.78%	9.49%	10.26%	7.80%	10.61%	6.76%							
Statin	No	246	35.58%	48.32%	35.98%	11.00%	35.00%	56.36%	58.43%	44.17%	32.69%	48.92%	27.80%	66.18%	35.02%	35.57%	36.42%	46.10%	42.45%	25.68%	*	*	*	*	*	*	*
	Yes	392	64.42%	51.68%	64.02%	89.00%	65.00%	43.64%	41.57%	55.83%	67.31%	51.08%	72.20%	33.82%	64.98%	64.43%	63.58%	53.90%	57.55%	74.32%							
Number of Non-diabetic medications	0	97	14.72%	16.78%	12.55%	3.00%	9.00%	24.55%	31.46%	16.87%	13.46%	18.15%	12.14%	41.18%	14.20%	9.49%	12.88%	23.40%	17.96%	6.08%		*		*	*	*	*
	1	79	11.45%	15.44%	10.04%	13.00%	11.00%	15.45%	15.73%	11.66%	13.14%	11.69%	13.10%	25.00%	13.56%	7.51%	11.27%	16.31%	14.29%	6.08%							
	2	102	17.79%	10.07%	18.41%	15.00%	15.00%	19.09%	7.87%	16.56%	15.38%	12.62%	19.49%	5.88%	16.40%	18.18%	17.30%	11.35%	18.16%	8.78%							
	3	132	21.68%	17.45%	21.76%	29.00%	23.00%	18.18%	8.99%	20.25%	21.15%	18.77%	22.68%	14.71%	21.45%	21.34%	20.52%	21.28%	21.84%	16.89%							
	4	95	13.91%	18.12%	10.04%	23.00%	18.00%	14.55%	15.73%	16.26%	13.46%	15.08%	14.70%	5.88%	15.77%	16.21%	15.90%	11.35%	12.45%	22.97%							
	5	61	8.38%	13.42%	14.23%	8.00%	6.00%	7.27%	5.62%	7.06%	12.18%	9.85%	9.27%	5.88%	8.52%	11.86%	9.86%	8.51%	7.14%	17.57%							
	6	38	5.93%	6.04%	9.21%	5.00%	8.00%	0.91%	2.25%	5.83%	6.09%	6.15%	5.75%	1.47%	5.36%	7.91%	6.84%	2.84%	3.67%	13.51%							
	7	26	4.70%	2.01%	3.77%	4.00%	6.00%	0.00%	7.87%	4.60%	3.53%	5.85%	2.24%	0.00%	3.47%	5.93%	4.02%	4.26%	3.27%	6.76%							
	8	3	0.61%	0.00%	0.00%	0.00%	0.00%	0.00%	3.37%	0.61%	0.32%	0.92%	0.00%	0.00%	0.63%	0.40%	0.40%	0.71%	0.41%	0.68%							
	9	3	0.41%	0.67%	0.00%	0.00%	2.00%	0.00%	1.12%	0.31%	0.64%	0.31%	0.64%	0.00%	0.63%	0.40%	0.60%	0.00%	0.41%	0.68%							
	10	2	0.41%	0.00%	0.00%	0.00%	2.00%	0.00%	0.00%	0.00%	0.64%	0.62%	0.00%	0.00%	0.00%	0.79%	0.40%	0.00%	0.41%	0.00%							

ACEI, Angiotensin-converting enzyme inhibitors; ARB, Angiotensin receptor agonist; ASCVD, Atherosclerotic cardiovascular disease; BP, Blood pressure; CCB, Calcium channel blockers; Con, controlled; CR, Central region; DC, Diabetes center; DM, Diabetes mellitus; DPP-4 inhibitors, Dipeptidyl-peptidase-4 inhibitors; ER, Eastern region; GLP-1 agonists, glucagon-like peptide-1 agonist; H, Hospital; NR, Northern region; SGLT-2 inhibitors, Sodium-glucose co-transporter-2 inhibitors; SR, Southern region; Uncon, Uncontrolled; WR, Western region.

This table shows the types of non-diabetic medication used by participants and the percentage of each type, categorized by diabetes control, region, gender, age, hospital type, blood pressure control and the presence or absence of atherosclerotic disease.

#### 3.4.3 Supplementation

The number of vitamins prescribed ranged from 0 to 5, the most common being a single supplement for 50% of patients. Vitamin B-complex, multivitamins, vitamin D, calcium carbonate, vitamin B12, folic acid, and Iron were the most common supplements recorded for the 638 participants in our study. Regions and gender were the most correlated variables with vitamins. Iron, folic acid, and vitamin D correlated more with the female group, while vitamin B12 correlated more with the male group (**Table 10**). The Vitamin B complex had the highest prescription rate in the Northern region (61%) vs. 29% in the Southern region. Dispensing of vitamin B complex was higher in diabetic centers than in hospitals (50% and 39%, respectively). Multivitamins were more prescribed in the Southern region (44%) vs. 17% in the Central region. Multivitamins prescriptions were higher in general hospitals than in diabetes centers (23% and 13%, respectively). Vitamin D was much more prescribed in the Northern region (40%) vs. the lowest in the Eastern region (12%). Using calcium was higher in the female group (7%) vs. 3% in the male group. Patients without ASCVD had higher vitamin D and B complex use than the ASCVD group.

**Table 10:** Types of supplements used by participants.

Supplements		Total	DM Control		Region					Gender		Hospital Type		Age			BP Control		ASCVD		DM	R	G	T	A	BP	D
			UnCon	Con	CR	WR	NR	SR	ER	M	F	H	DC	20-41	41-60	>60	UnCon	Con	No	Yes	* denotes P value < 0.05						
		Total	489	149	239	100	100	110	89	326	312	325	313	68	317	253	497	141	490	148							
Vitamin B complex	No	353	53.8%	60.4%	52.7%	58.0%	39.0%	70.9%	58.4%	58.9%	51.6%	60.9%	49.5%	45.6%	58.7%	53.8%	54.1%	59.6%	52.7%	64.2%		*		*			*
	Yes	285	46.2%	39.6%	47.3%	42.0%	61.0%	29.1%	41.6%	41.1%	48.4%	39.1%	50.5%	54.4%	41.3%	46.2%	45.9%	40.4%	47.3%	35.8%							
Multivitamins	No	518	80.4%	83.9%	82.4%	88.0%	84.0%	55.5%	98.9%	82.2%	80.1%	76.3%	86.3%	86.8%	78.5%	83.0%	80.7%	83.0%	81.8%	79.1%		*		*			
	Yes	120	19.6%	16.1%	17.6%	12.0%	16.0%	44.5%	1.1%	17.8%	19.9%	23.7%	13.7%	13.2%	21.5%	17.0%	19.3%	17.0%	18.2%	20.9%							
Vitamin D	No	491	77.5%	75.2%	72.8%	72.0%	60.0%	97.3%	87.6%	79.8%	74.0%	79.7%	74.1%	77.9%	77.0%	76.7%	76.7%	78.0%	74.9%	83.8%		*					*
	Yes	147	22.5%	24.8%	27.2%	28.0%	40.0%	2.7%	12.4%	20.2%	26.0%	20.3%	25.9%	22.1%	23.0%	23.3%	23.3%	22.0%	25.1%	16.2%							
Calcium carbonate	No	604	95.7%	91.3%	91.2%	95.0%	98.0%	97.3%	96.6%	96.6%	92.6%	95.1%	94.2%	97.1%	96.2%	92.1%	95.0%	93.6%	95.1%	93.2%	*	*	*				
	Yes	34	4.3%	8.7%	8.8%	5.0%	2.0%	2.7%	3.4%	3.4%	7.4%	4.9%	5.8%	2.9%	3.8%	7.9%	5.0%	6.4%	4.9%	6.8%							
Vitamin B12	No	593	95.5%	84.6%	99.2%	70.0%	100.0%	100.0%	85.4%	90.8%	95.2%	95.4%	90.4%	98.5%	91.2%	93.7%	94.6%	87.2%	93.5%	91.2%	*	*	*	*		*	
	Yes	45	4.5%	15.4%	0.8%	30.0%	0.0%	0.0%	14.6%	9.2%	4.8%	4.6%	9.6%	1.5%	8.8%	6.3%	5.4%	12.8%	6.5%	8.8%							
Folic acid	No	606	95.7%	92.6%	94.1%	99.0%	93.0%	95.5%	94.4%	96.9%	92.9%	92.0%	98.1%	97.1%	96.8%	92.1%	96.0%	91.5%	95.5%	93.2%			*	*	*	*	
	Yes	32	4.3%	7.4%	5.9%	1.0%	7.0%	4.5%	5.6%	3.1%	7.1%	8.0%	1.9%	2.9%	3.2%	7.9%	4.0%	8.5%	4.5%	6.8%							
Sum	0	162	25.15%	26.17%	29.29%	14.00%	13.00%	25.45%	41.57%	29.14%	21.47%	29.54%	21.09%	29.41%	24.61%	25.30%	24.35%	29.08%	23.27%	32.43%	*	*	*			*	
	1	319	50.72%	47.65%	44.77%	55.00%	50.00%	64.55%	40.45%	50.92%	49.04%	45.54%	54.63%	45.59%	52.68%	47.83%	53.12%	39.01%	51.63%	44.59%							
	2	118	19.43%	15.44%	15.48%	29.00%	30.00%	8.18%	14.61%	16.87%	20.19%	17.85%	19.17%	20.59%	18.93%	17.39%	16.90%	24.11%	18.78%	17.57%							
	3	29	4.09%	6.04%	6.69%	2.00%	6.00%	1.82%	3.37%	2.15%	7.05%	5.23%	3.83%	4.41%	3.15%	6.32%	4.63%	4.26%	4.90%	3.38%							
	4	7	0.61%	2.68%	2.51%	0.00%	1.00%	0.00%	0.00%	0.31%	1.92%	0.92%	1.28%	0.00%	0.32%	2.37%	0.40%	3.55%	1.02%	1.35%							
	5	3	0.00%	2.01%	1.26%	0.00%	0.00%	0.00%	0.00%	0.61%	0.32%	0.92%	0.00%	0.00%	0.32%	0.79%	0.60%	0.00%	0.41%	0.68%							

ASCVD, Atherosclerotic cardiovascular disease; BP, Blood pressure; Con, controlled; CR, Central region; DC, Diabetes center; DM, Diabetes mellitus; ER, Eastern region; H, Hospital; NR, Northern region; SR, Southern region; Uncon, Uncontrolled; WR, Western region.

The above table displays the types of supplements used by participants and the percentage of each type, categorized by diabetes control, region, gender, age, hospital type, blood pressure control and the presence or absence of atherosclerotic disease.

### 3.5 Complications and comorbidities

#### 3.5.1 Complications

Results of complications showed that the highest share, with 65% of the participants, was for patients with no complications, the second largest share with 23% for a single complication, and the rest 12% had more than two complications. The most common complications were cerebrovascular disease (i.e., TIA, stroke), coronary artery disease (i.e., MI, stable angina), nephropathy (Including ESRD), peripheral neuropathy ( i.e., foot infection, amputation), autonomic neuropathy (i.e., erectile dysfunction, gastroparesis, loss of bladder control, urinary tract infection), and retinopathy. Statistical results showed that regions followed by ASCVD status were the most deterministic complications.

Nephropathy was recorded in 53 patients. The highest rate was recorded in the western region, with 20%, while the lowest was in the Southern part, with no records of nephropathy. The nephropathy rate in the male group was higher than in the female group (11% for males vs. 4% for females).

Peripheral neuropathy was recorded in 69 patients, while the highest rate was uncontrolled T2DM vs. controlled T2DM (12% vs. 5%). The highest peripheral neuropathy was observed in the Western region (22% of patients) vs. 7% in the Northern region. Similarly, the male group was higher than the female group (14% vs. 7%, respectively).

Retinopathy was recorded in 46% of the western region, followed by the Eastern 20%, and there were no records of patients with retinopathy in the Northern region. The male group showed higher rates of retinopathy than the female group (**Table 11**).

**Table 11:** Types of complications experienced by participants.

Complications		Total	DM Control		Region					Gender		Hospital Type		Age			BP Control		ASCVD		DM	R	G	T	A	BP	D
			UnCon	Con	CR	WR	NR	SR	ER	M	F	H	DC	20-41	41-60	>60	UnCon	Con	No	Yes	* denotes P value < 0.05						
		Total	489	149	239	100	100	110	89	326	312	325	313	68	317	253	497	141	490	148							
Cerebrovascular disease (TIA, stroke….)	No	614	96.3%	96.0%	91.6%	100.0%	99.0%	100.0%	96.6%	95.4%	97.1%	93.8%	98.7%	100.0%	96.5%	94.9%	96.4%	95.7%	99.4%	85.8%		*		*			*
	Yes	24	3.7%	4.0%	8.4%	0.0%	1.0%	0.0%	3.4%	4.6%	2.9%	6.2%	1.3%	0.0%	3.5%	5.1%	3.6%	4.3%	0.6%	14.2%							
Coronary artery disease (MI, stable angina….)	No	589	92.4%	91.9%	90.4%	100.0%	95.0%	95.5%	82.0%	90.5%	94.2%	88.3%	96.5%	100.0%	93.4%	88.9%	93.2%	89.4%	99.2%	69.6%		*		*	*		*
	Yes	49	7.6%	8.1%	9.6%	0.0%	5.0%	4.5%	18.0%	9.5%	5.8%	11.7%	3.5%	0.0%	6.6%	11.1%	6.8%	10.6%	0.8%	30.4%							
Nephropathy (Include ESRD)	No	585	92.6%	88.6%	91.6%	80.0%	93.0%	100.0%	93.3%	88.3%	95.2%	92.6%	90.7%	98.5%	92.7%	88.5%	91.5%	92.2%	93.1%	87.2%		*	*		*		*
	Yes	53	7.4%	11.4%	8.4%	20.0%	7.0%	0.0%	6.7%	11.7%	4.8%	7.4%	9.3%	1.5%	7.3%	11.5%	8.5%	7.8%	6.9%	12.8%							
Peripheral neuropathy (Foot infection, amputation….)	No	569	87.5%	94.6%	92.5%	78.0%	94.0%	90.0%	86.5%	85.6%	92.9%	88.9%	89.5%	97.1%	87.7%	88.9%	89.7%	87.2%	90.0%	86.5%	*	*	*				
	Yes	69	12.5%	5.4%	7.5%	22.0%	6.0%	10.0%	13.5%	14.4%	7.1%	11.1%	10.5%	2.9%	12.3%	11.1%	10.3%	12.8%	10.0%	13.5%							
Retinopathy	No	544	85.9%	83.2%	90.8%	54.0%	100.0%	92.7%	79.8%	82.2%	88.5%	91.7%	78.6%	92.6%	86.1%	82.2%	86.9%	79.4%	85.9%	83.1%		*	*	*		*	
	Yes	94	14.1%	16.8%	9.2%	46.0%	0.0%	7.3%	20.2%	17.8%	11.5%	8.3%	21.4%	7.4%	13.9%	17.8%	13.1%	20.6%	14.1%	16.9%							
Number of complications	M	417	64.6%	67.8%	64.4%	45.0%	81.0%	77.3%	58.4%	59.2%	71.8%	65.2%	65.5%	88.2%	66.9%	57.3%	67.4%	58.2%	73.9%	37.2%		*	*		*		*
	1	148	24.1%	20.1%	26.8%	28.0%	17.0%	20.0%	19.1%	24.8%	21.5%	22.8%	23.6%	8.8%	22.1%	28.5%	21.9%	27.7%	18.4%	39.2%							
	2	54	8.4%	8.7%	6.3%	21.0%	2.0%	2.7%	14.6%	11.0%	5.8%	8.3%	8.6%	2.9%	7.6%	11.1%	7.8%	10.6%	5.9%	16.9%							
	3	14	2.0%	2.7%	2.1%	5.0%	0.0%	0.0%	4.5%	3.7%	0.6%	2.5%	1.9%	0.0%	2.2%	2.8%	2.4%	1.4%	1.6%	4.1%							
	4	5	0.8%	0.7%	0.4%	1.0%	0.0%	0.0%	3.4%	1.2%	0.3%	1.2%	0.3%	0.0%	1.3%	0.4%	0.4%	2.1%	0.2%	2.7%							

ASCVD, Atherosclerotic cardiovascular disease; BP, Blood pressure; Con, controlled; CR, Central region; DC, Diabetes center; DM, Diabetes mellitus; ER, Eastern region; ESRD, End stage renal disease; H, Hospital; MI, Myocardial Infraction; NR, Northern region; SR, Southern region; TIA, transient ischemic attack; Uncon, Uncontrolled; WR, Western.

This table shows the complications experienced by participants and the percentage of each complication, categorized by diabetes control, region, gender, age, hospital type, blood pressure control and the presence or absence of atherosclerotic disease.

#### 3.5.2 Comorbidities

The most common comorbidities observed were ASCVD, neuropathy, COPD, prostate hyperplasia, hypertension (HTN), and thyroid disorders. Other comorbidities, i.e., cataracts, CKD, conjunctivitis, disorder of the cornea, erectile dysfunction, gastroenteritis and colitis, hypercholesterolemia, interstitial lung disease, and obstructive sleep apnea syndrome, were excluded as they were recorded in less than 10 participants. The number of comorbidities ranged from 0 to 8, with the largest share for single comorbidities followed by no or missing comorbidities. The most common comorbidities in uncontrolled and controlled T2DM patients were hypertension, followed by ASCVD. Statistical results showed that regions and hospital types were the most correlated factors with different comorbidities. The number of patients with hypertension in the female group was 61% vs. 52% in the male group. In the Western region, a 70% rate of hypertension was highest vs. the Eastern region at 48%. The patients’ rates of hypertension in diabetic centers were higher than in general hospitals (60% vs. 52%, respectively). Results showed the highest age group with hypertension was between 40-60, while the age group over 60 had the highest coronary artery diseases with 11%, while no coronary artery disease was recorded among the 20–40 age group.

The highest level of thyroid disorders was recorded in the Northern region, with 18%, while the lowest was recorded in the Southern region, with 3%. The female group was three times higher in thyroid disorders vs. the male group (**Table 12**).

**Table 12:** Types of comorbidities experienced by participants.

Comorbidities		Total	DM Control		Region					Gender		Hospital Type		Age			BP Control		ASCVD		DM	R	G	T	A	BP	D
			UnCon	Con	CR	WR	NR	SR	ER	M	F	H	DC	20-41	41-60	>60	UnCon	Con	No	Yes	* denotes P value < 0.05						
		Total	489	149	239	100	100	110	89	326	312	325	313	68	317	253	497	141	490	148							
Neuropathy	No	602	95.50%	90.60%	94.56%	93.00%	96.00%	95.45%	92.13%	94.17%	94.55%	92.92%	95.85%	98.53%	94.64%	92.89%	95.17%	91.49%	94.29%	94.59%	*						
	Yes	36	4.50%	9.40%	5.44%	7.00%	4.00%	4.55%	7.87%	5.83%	5.45%	7.08%	4.15%	1.47%	5.36%	7.11%	4.83%	8.51%	5.71%	5.41%							
Hypertension (HTN)	No	278	42.33%	47.65%	45.61%	30.00%	49.00%	40.00%	51.69%	47.55%	39.42%	47.38%	39.62%	76.47%	44.16%	33.99%	39.24%	58.87%	47.14%	31.76%		*	*	*	*	*	*
	Yes	360	57.67%	52.35%	54.39%	70.00%	51.00%	60.00%	48.31%	52.45%	60.58%	52.62%	60.38%	23.53%	55.84%	66.01%	60.76%	41.13%	52.86%	68.24%							
Thyroid disorders	No	568	91.62%	80.54%	90.38%	89.00%	82.00%	96.36%	84.27%	93.25%	84.62%	88.92%	89.14%	86.76%	89.91%	88.54%	88.13%	92.20%	87.76%	93.24%	*	*	*				
	Yes	70	8.38%	19.46%	9.62%	11.00%	18.00%	3.64%	15.73%	6.75%	15.38%	11.08%	10.86%	13.24%	10.09%	11.46%	11.87%	7.80%	12.24%	6.76%							
Number of complications	0	156	24.34%	24.83%	27.20%	11.00%	19.00%	32.73%	28.09%	28.83%	19.87%	26.15%	22.68%	51.47%	26.81%	14.23%	21.53%	34.75%	28.78%	10.14%		*		*	*	*	*
	1	243	39.47%	33.56%	41.00%	32.00%	49.00%	35.45%	28.09%	35.58%	40.71%	36.62%	39.62%	35.29%	38.17%	38.74%	40.04%	31.21%	39.39%	33.78%							
	4	9	1.43%	1.34%	0.00%	2.00%	0.00%	1.82%	5.62%	1.53%	1.28%	2.15%	0.64%	0.00%	0.95%	2.37%	1.01%	2.84%	1.63%	0.68%							
	5	8	1.43%	0.67%	0.00%	0.00%	0.00%	0.00%	8.99%	1.53%	0.96%	2.46%	0.00%	0.00%	0.32%	2.77%	0.80%	2.84%	1.22%	1.35%							
	6	4	0.82%	0.00%	0.00%	0.00%	0.00%	0.00%	4.49%	0.00%	1.28%	1.23%	0.00%	0.00%	0.95%	0.40%	0.80%	0.00%	0.00%	2.70%							
	7	1	0.20%	0.00%	0.00%	0.00%	0.00%	0.00%	1.12%	0.00%	0.32%	0.31%	0.00%	0.00%	0.00%	0.40%	0.20%	0.00%	0.20%	0.00%							
	8	3	0.20%	1.34%	0.00%	0.00%	0.00%	0.00%	3.37%	0.61%	0.32%	0.92%	0.00%	0.00%	0.32%	0.79%	0.40%	0.71%	0.61%	0.00%							

ASCVD, Atherosclerotic cardiovascular disease; BP, Blood pressure; Con, controlled; CR, Central region; DC, Diabetes center; DM, Diabetes mellitus; ER, Eastern region; H, Hospital; NR, Northern region; SR, Southern region; Uncon, Uncontrolled; WR, Western region.

This table presents the comorbidities experienced by participants, along with the percentage of each comorbidity, categorized by diabetes control, region, gender, age, hospital type, blood pressure control and the presence or absence of atherosclerotic disease.

## 4 Discussion

### 4.1 Prevalence and uncontrolled diabetes and BP

#### 4.1.1 Nationwide

Several previous studies have investigated T2DM status in KSA, some of which were limited to a single or few cities (11, 21–23), and only a few studies have investigated the T2DM status nationwide (24–26). We have investigated the nationwide status of T2DM in KSA’s five administrative regions. Previous studies have primarily focused on the Central, Southern, and Western regions, and only a few have included the Northern and Eastern regions (11, 12, 21, 27).

### 4.2 T2DM control

Alsuliman et al. (2021) reported that Individuals with T2DM in KSA had a pooled prevalence of 77.7% for poor glycemic control, which is in agreement with our study (77%) (28). AlMutairi et al. (2013) reported that 74% of respondents had poor glycemic control among selected samples from AlMadinah (29). According to the Saudi MOH, the highest percentage of T2DM prevalence was reported from Hail (Northern region), while the lowest was from Jazan (Southern region) (30). The lowest prevalence of T2DM was reported from the Southern region (30); our data showed the highest controlled percentage among patients from the Eastern and Western regions, with 40% controlled patients and the lowest was reported from the Northern and Central regions. Al-Ghamdi et al. (2004) reported 77% poor control from Jeddah (Western region), and this discrepancy may be attributed to the inclusion of T1DM and T2DM patients in their study (31). Our results showed HbA1c levels ranged from 5 to 16.3% with an average of 8.6 ± 0.07% in all regions, which indicates an uncontrolled DM situation and is consistent with other studies. AlRubaiaan et al. (2020) reported an average HbA1c of 8.8 ± 1.7%, and Alramadan et al., 2017 reported that HbA1c levels were 8.20 ± 1.89 in the largest three cities in KSA (24, 32). Our results showed no significant differences among all regions of KSA.

### 4.3 Hypertension

Almalki et al. (2020) reported that 71.8% of the 1178 subjects had uncontrolled hypertension (33), by 77.9% of diabetics in all regions had uncontrolled hypertension in our data. This is also consistent with Charbel El Bcheraoui et al. (2013), who reported high rates of hypertension and borderline hypertension in KSA. They also reported high rates of uncontrolled hypertension in KSA (34). Our data showed that hypertension at 79%, followed by ASCVD at 75%, were the most common comorbidities in T2DM patients in all regions of KSA. On the contrary, Einarson et al. (2018) showed that the prevalence of cardiovascular disease in T2DM patients affects approximately 32.2% of the population worldwide (35). Nielsen reported that hypertension among T2DM patients was 19% in the Saudi population (36). The low percentage reported by Nielsen could be explained by the definition of hypertension reported in the article with levels above 160/95mmHg. On the other hand, according to the AHA, we have reported patients above 130/80 mmHg to be hypertensive (37). The highest BP was in the Western region (70%), while the lowest region was in the Eastern region (48%). This is consistent with Al-Nozha et al. (1998), who conducted the study in all Saudi regions and reported that the prevalence of systolic BP was higher in Taif, Farasan, and Hail while it was lower in Asir, Jizan, and Al Madinah (26).

### 4.4 Age and gender

We observed no significant difference between male and female groups in T2DM control with around 70-80% uncontrolled patients. Similar results have been reported by Alramadan et al. (2018) from three major cities, i.e., Hofuf, Riyadh, and Jeddah. Al-Hazmi et al. (1995) reported in their nationwide study slight differences between male and female groups (25). Alramadan et al. (2018) have reported a high prevalence of T2DMZ among older people (>60 years) age group (23). In the present study, no differences were observed among different age groups.

### 4.5 Medications

Few studies have investigated patients’ medication nationwide (32, 38). The present study has investigated diabetes patients’ medication on anti-diabetes, non-diabetes, and Supplementation drugs.

The number of anti-hyperglycemic used for all patients was three in over 30% of our sample. In comparison, over 40% of patients used one or two drugs, including biguanides, DDP-4 inhibitors, GLP-1 agonists, Insulin, SGLT-2 inhibitors, and sulfonylurea. Badedi et al. (2016) reported that the average number of drugs used to control T2DM in the primary center in Jazan City was four (39). According to Grant et al. (2003), drugs for treating T2DM patients in the USA were four on average from different antihyperglycemic groups (40). ADA guidelines recommended metformin as the first-line treatment for T2DM patients (41). Al-Rubeaan et al. (2020) reported that metformin is the most prescribed first-line treatment for patients with T2DM from Central, Northern, Southern, and Western regions, managed either in governmental institutions or in the private sector in representative hospitals either alone or in combination with different medications in 50% of cases (32). Our results agree with Al-Rubeaan’s study that metformin was the most prescribed medication in all regions (32). Moreover, metformin was most prescribed in the Western and Eastern regions. At the same time, the lowest rate was found in the Northern and Central regions, with 15% differences between the two groups. Notably, regions with higher metformin prescriptions were found to have higher rates of controlled patients.

Al-Rubeaan et al. reported that sitagliptin was the second most frequently used in T2DM treatment (32). DPP-4 was our study's second most commonly prescribed drug (47%) after metformin. This is consistent with Saudi Diabetes Clinical Practice (SDCP) and ADA Guidelines. ADA guidelines recommended sodium–glucose cotransporter 2 (SGLT-2) inhibitor or glucagon-like peptide 1 (GLP-1) receptor agonist for T2DM patients who have established ASCVD or have indicators of high-risk, established kidney disease, or HF, with demonstrated CVD benefit is recommended as part of the glucose-lowering regimen independent of A1C and in consideration of patient-specific factors (19, 41). Our findings showed that GLP 1 agonists were prescribed the most in Western and Eastern regions, in line with the lowest coronary artery disease rate among all regions.

Besides ADA guidelines, the SDCP Guidelines 2021 also recommended SGLT-2 inhibitors as one of the options after metformin because of their cardiovascular and kidney protection characteristics (19, 41). Eastern and Western regions again had the highest SGLT-2 inhibitors prescription rate. European Society of Cardiology (ESC) and, American College of Cardiology (ACC), Saudi Heart Association (SHA) guidelines recommend SGLT-2 inhibitors for patients with cardiovascular ailments (37, 41, 42).

Al-Rubeaan et al. clarified the reasons for changing first-line antidiabetic drugs to be a lack of efficacy or secondary failure of oral medications in achieving HbA1c target levels (41). ADA has recommended Insulin for patients who failed to achieve target levels of HbA1c with oral antihyperglycemic drugs (41). Zhang et al. (2022) suggested that insulin therapy should be added to the HbA1c control regimen to prevent complications of T2DM (43). Khunti et al. (2016) reported that insulin was primarily used with patients with poor glycemic control (44). Our study showed that insulin intake was higher among uncontrolled patients and those with ASCVD episodes. The Southern region showed the highest insulin intake rate (70%), while the Eastern region had the lowest (38%).

### 4.6 Non-diabetes medications

The present study is the first to investigate non-diabetes medication usage besides antihyperglycemic drugs in KSA. The number of drugs recorded in the present study was up to ten (averaging from one to four). Al-Osaimi et al. (2022) reported that around thirty percent of chronic patients took four to six medications daily in KSA (45). ADA, ESC, SDCP, ACC, and Saudi hypertension Guidelines recommend ACEI or ARB for T2DM patients (19, 41, 46–48). Our results showed that only 45% of T2DM patients were on ACEI or ARB, while 77.9% of patients with uncontrolled hypertension were not receiving treatment according to the recommended Guidelines.

Beta-blockers were prescribed for patients over 60 and with ASCVD. This is consistent with ESC recommendations for beta-blocker intake for heart failure and myocardial infarction patients (41, 49). Our results showed that the highest CCB usage was observed in hypertensive T2DM patients in the Central region and lowest in the Southern region. International Society of Hypertension Global Hypertension Practice Guidelines recommended ACEis/ARBs and CCBs as the first-line treatment for patients with hypertension and T2DM (50). This is also in accordance with the British and Irish Hypertension Society and National Institute for Health and Care Excellence (NICE) guidelines (51, 52).

ADA and AHA Guidelines highly recommend statin therapy for all T2DM patients, whether in high or moderate intensity, depending on the patient’s condition (41, 53). Statins were prescribed for almost half of our sample. The results also showed that diabetes centers prescribe more statins than general hospitals and that statins are more commonly prescribed with ASCVD than those without, which agrees with the ADA Guidelines. ADA Guidelines recommended that patients with ASCVD or at high risk of ASCVD should be on antiplatelet as a secondary preventive measure and for T2DM patients as prophylaxis (41).

### 4.7 Supplements

Our study is the first to investigate supplement drugs used with antihyperglycemic drugs in all regions of KSA. There were six leading supplements for T2DM patients. ADA Guidelines recommend vitamin B12 intake for T2DM patients to enhance their quality of life, especially those on metformin (41). Our results showed that Vitamin B12 intake was higher among males than females. It was also prescribed more in diabetic centers vs. general hospitals (50% and 39%, respectively). On the contrary, Iron, folic acid, and vitamin D were more correlated in the female and male groups. This may be because women have these supplements during pregnancy. This is consistent with Guillaume et al., who reported that two-thirds of pregnant women received iron supplementation during pregnancy (54). Additionally, WHO has recommended folic acid (FA) supplementation for pregnant women to prevent anemia and fetal complications (55). Sulaiman et al. reported high vitamin D deficiency among female university students in Northern KSA (56).

### 4.8 Complications and comorbidities

The present study is one of the few to report complications and comorbidities among T2DM patients in all general hospitals and diabetes centers across the kingdom. T2DM complications may even exist at the early stages of the disease. Our data showed complications ranging from one to six among all T2DM patients. Recorded complications include retinopathy, peripheral neuropathy, nephropathy, coronary artery disease, autonomic neuropathy (foot infection and amputation), and cerebral vascular disease. Alshaya et al. reported six major complications, i.e., peripheral neuropathy, dyslipidemia, retinopathy, nephropathy, and diabetic feet, among T2DM patients in the Northern region (57). Alhammadi et al. reported that the five most common complications among T2DM patients in the Aseer region included retinopathy 31.5%, followed by coronary artery disease 23.2%, nephropathy 19.5%, diabetic feet 15.5%, and stroke14.8% (58). Sulimani et al. reported that the most common complications were peripheral vascular disease at 54.5%, followed by peripheral neuropathy at 48.8% (59). Ziaul et al., 2019 have recorded retinopathy amongst T2DM patients of the Central region (60). Abdulghani et al., 2018 reported a wide range of complications in the Central region that included dyslipidemia (58.6%), retinopathy (23.3%), heart disease (14.4%), and severe foot complications (3.9%) (61).

### 4.9 Comorbidities

Our results showed that the number of comorbidities ranged from zero to eight, with the largest share for single comorbidity. Our Results showed that the most common comorbidities within uncontrolled and controlled diabetes patients were hypertension, ASCVD, Neuropathy, thyroid disorder, and COPD. Akin et al. (2020) reported that hypertension is the most common comorbidity among diabetes patients, with 84.9% in Turkey (62). Alshaya et al., 2017) obtained similar results, with 56 % in the Northern region of KSA. (58), Abdulghani et al., 2018 with 61.4% in the central region of KSA, and Iglay et al., 2017 with 82.1% in Jazan City in the Southern region of KSA (61, 63).

Results showed that the number of patients with hypertension in the female group was slightly higher than in the male group among the uncontrolled patients. This is different from the study done by Al-Nozha et al., 2007 who reported that the prevalence of hypertension in males was 28.6%, against only 23.9% in the female group (64). Our results showed that ASCVD was recorded for around 30% of patients across the whole kingdom. Fatani et al., 1989 reported that ASCVD in diabetic patients was 11.1% in the western region (65). Thyroid disorders were recorded in 11% of diabetic patients. This is consistence with results obtained by Hammadi et al., 2018 that thyroid disorders are pretty high among T2D patients in the Western region (66).

The strength of this study is that it covers the comprehensive inclusion of data from multiple administrative regions in KSA, giving a robust epidemiological overview of diabetes management. A limitation of this study is that patients with orthostatic hypotension-a sign of cardiac autonomic neuropathy-were excluded, which might have influenced findings related to cardiovascular complications in T2DM patients. Future studies should try to include cases like this to have a more specific knowledge of diabetes complications.

## 5 Conclusion

Enhancing healthcare quality in the Kingdom is the primary goal of KSA Vision 2030 by improving the quality of preventive and therapeutic healthcare services. Improving treatment for chronic diseases, including diabetes, remains the health policy's cornerstone. Our results showed that 77% of our sample have uncontrolled diabetes, which should alarm the health authorities to take serious measures to stop the disease progression and avoid other complications, i.e., retinopathy, peripheral neuropathy, and comorbidities, i.e., hypertension and ASCVD. Most of those affected by diabetes are between the age of 40 to 60. Although life expectancy in KSA has increased to 75 years, preventive measures would save the country healthcare costs, time, and effort to treat. Variation among regions was evident in our study. Still, further investigations could be conducted with a larger sample size to confirm our findings. Our data show regions with the highest T2DM control rates were higher in metformin intake, followed by GLP-1 and SGLT-2, which align with ADA Guidelines. ADA, ESC, ACC, and SHA Guidelines recommend the intake of ACE/ARBs for T2DM patients with hypertension or ASCVD and kidney protection. Our results showed ACE/ARBs intake variations among different regions and age groups. Our results showed significant variation among diabetes patients regarding medications that necessitate Guideline unification and implementation, whether for anti-diabetes or non-diabetes drugs and supplements.

Generally, we observed suboptimal adherence to many ADA Guidelines to treat T2DM patients at diabetes centers and general hospitals which used similar treatment protocols. However, diabetes centers were unique in using thyroxine and vitamin B complex prescriptions. However, despite recommendations for T2DM patients, diabetes centers outperform general hospitals in the use of statins which shows the importance of diabetes centers in comparison to public hospitals in T2DM treatment.

Monitoring programs on the national level with a larger sample size and broader distribution should be conducted every five years to assess disease progression and the efficacy of the implemented treatment strategies. There is an urgent need for more documentation, a critical tool for monitoring disease progression nationally. Physicians and Pharmacists’ awareness and training are of great significance, and strategies should be developed to achieve the kingdom’s goal of fighting diabetes and improving T2DM patients’ quality of life.

## Data Availability

The original contributions presented in the study are included in the article/supplementary material. Further inquiries can be directed to the corresponding author.
